# Levels of 17β-Estradiol Receptors Expressed in Embryonic and Adult Zebrafish Following *In Vivo* Treatment of Natural or Synthetic Ligands

**DOI:** 10.1371/journal.pone.0009678

**Published:** 2010-03-12

**Authors:** Gayathri Chandrasekar, Amena Archer, Jan-Åke Gustafsson, Monika Andersson Lendahl

**Affiliations:** Department of Biosciences and Nutrition, Karolinska Institutet, Stockholm, Sweden; Institute of Genetics and Molecular and Cellular Biology, France

## Abstract

The nuclear receptors encompass a group of regulatory proteins involved in a number of physiological processes. The estrogen receptors (ERs), of which one alpha and one beta form exist in mammals function as transcription factors in response to 17β-estradiol (E2). In zebrafish there are three gene products of estrogen receptors and they are denoted *esr1* (ERalpha), *esr2a* (ERbeta2) and *esr2b* (ERbeta1). Total RNA of zebrafish early life stages (<3, 6, 12, 24, 48, 72, 96 and 120 hours post fertilization) and of adult fish (liver, intestine, eye, heart, brain, ovary, testis, gill, swim bladder and kidney) were isolated following in vivo exposures. Using specific primers for each of the three zebrafish ERs the expression levels were quantified using real time PCR methodology. It was shown that in absence of exposure all three estrogen receptors were expressed in adult fish. The levels of expression of two of these three ER genes, the *esr1* and *esr2a* were altered in organs such as liver, intestine, brain and testis in response to ligand (E2, diethylstilbestrol or 4-nonylphenol). During embryogenesis two of the three receptor genes, *esr1* and *esr2b* were expressed, and in presence of ligand the mRNA levels of these two genes increased. The conclusions are i) estrogen receptor genes are expressed during early development ii) altered expression of *esr* genes in response to ligand is dependent on the cellular context; iii) the estrogenic ligand 4-nonylphenol, a manufactured compound commonly found in sewage of water treatment plants, acts as an agonist of the estrogen receptor during development and has both agonist and antagonist properties in tissues of adult fish. This knowledge of *esr* gene function in development and in adult life will help to understand mechanisms of interfering mimicking endocrine chemicals *in vivo*.

## Introduction

The cDNAs coding for the alpha and beta forms of the receptor proteins that bind to 17β-estradiol (E2) were isolated and cloned in the 1980ies and in the 1990ies [1]–[5]. ERalpha and ERbeta belong to the large family of nuclear receptors and they function as transcription factors following binding to their ligands [6]. The diverse cellular functions of the two receptors have been characterized in cell lines and with mouse ER knock out technology [7]–[10]. The sequence conservation between the human ERs and the corresponding receptors in other species is high, in particular within the domains of DNA binding (DBD) and ligand binding (LBD).

In contrast to mammalian species many of the fish species harbor three estrogen receptor genes, one alpha and two beta types [11], [12]. In zebrafish these receptor genes are designated *esr1* (ERalpha), *esr2b* (ERbeta1) and *esr2a* (ERbeta2) and it has been shown that the ligand 17β-estradiol binds to all three receptors with similar affinities as to the corresponding mammalian receptors [13], [14]. These ERs also have capacity to bind a large number of synthetic ligands [13], [15] with affinities similar to that of the natural ligand or for some ligands a thousand fold less than that for 17β-estradiol. Some synthetic estrogens have been detected in river waters [16] and at least one of these ligands, the nonylphenol (4-NP) is frequently found in sewage of water treatment plants [17] of the industrial world.

In an aim to evaluate ER expression and regulation in the zebrafish system we used E2 and two synthetic ligands, diethylstilbestrol (DES) and 4-NP to establish a model in which we determined the levels of receptors in absence of ligand or following exposure to these ligands. The zebrafish ERalpha gene autoregulates itself in liver after exposure to hormone [18] and we therefore wanted to delineate the expression of *esr1*, and the two beta subtypes of the estrogen receptor genes as possible targets of estrogenic ligands. This was done in embryos, in early larvae and in organs of adult zebrafish. To compare possible variations of the expression levels in organs of adult fish, RNAs of individual sibling fish were isolated and tested.

We show that the onset of *esr1*, *esr2b* and *esr2a* expression differs during embryogenesis. During development two out of three ERs, the *esr1* and the *esr2b* are well expressed while *esr2a* represents high levels before, and low levels beyond midblastula transition (MBT). In adult fish all three ERs are expressed in the organs tested except for the very low levels detected in ovaries. The effects on ER expression as a result from exposures of natural or synthetic ligands are discussed.

## Materials and Methods

### Ethics statement

All experiments are pursued according to the national guidelines and have been approved of by the local ethical committee Stockholms södra djurförsöksetiska nämnd (S-200-06 and S-1-10).

### Zebrafish maintenance

Adult zebrafish were maintained in 15 L aquaria at 28°C (Schwartz) supplied continuously with reverse osmosis water under 14 h of light and 10 h of dark cycle. Zebrafish embryos were raised, maintained and staged as previously described by Kimmel et al [19]. Staging of embryos is denoted as hours post fertilization (hpf).

### Embryos and tissues

Tissues from untreated adult males and females (Tűbingen strain, 12 to 18 months old) including brain, eye, gill, heart, liver, intestine, swim bladder, kidney, testis and ovary were dissected out and pooled by tissue type for RNA extraction. All tissues were stored at −80°C for subsequent RNA extraction. Embryos (untreated) were collected after spawning and allowed to develop in a Petri dish at 28°C. Embryos of different stages (<3, 6, 12, 24, 48, 72, 96 and 120 hpf) were collected, quickly frozen on dry ice and stored at −80°C until analyzed.

17β-estradiol (E2), 4-nonylphenol (4-NP) and diethylstilbestrol (DES) were purchased from Sigma. A 1 mM stock solution of E2, DES or 4-NP in 100% ethanol was diluted in embryo medium (5 mM NaCl, 0.17 mM KCl, 0.33 mM CaCl_2_, 0.33 mM MgSO_4_) to obtain 1 µM and 100 nM concentrations of E2, 4-NP and DES. Three to five adult fish (female and male) were exposed to 1 µM of ligand or vehicle (0.1% ethanol) for 48 h. After exposures, tissues were collected from individual organs, quickly frozen on dry ice and stored at −80°C. A clutch of zebrafish embryos, usually 200–300 embryos from a single pair of adult fish was divided and transferred into two Petri dishes, one containing the ligand and the other containing control embryos. Embryos were exposed to E2 (100 nM and 1 µM), DES (100 nM) or 4-NP (1 µM). Exposure of embryos was initiated one hour post fertilization and kept until day five at 28 degrees Celsius. Embryo media containing ligand were exchanged every second day. At different time points embryos were collected for RNA extractions.

### RNA extraction and cDNA synthesis

Total RNA from isolated organs of adult fish and from pooled embryos were extracted using Trizol reagent (Invitrogen) and RNeasy Mini Kit columns (Qiagen GmbH, Germany) according to the manufacturer's instructions. RNA concentrations were measured in a spectrophotometer and RNA integrity was analyzed by gel electrophoresis. Total RNA (1 µg) was treated with DNase I (amplification grade, Invitrogen) to remove DNA contaminations prior to cDNA synthesis carried out using Superscript II reverse transcriptase (Invitrogen). In the real-time PCR assays template cDNAs were diluted ten times.

### Real time PCR

Real-time PCR was pursued using Power SYBR Green master mix (Applied Biosystems) and amplified using an ABI Prism 7500 Sequence detector (Applied Biosystems). Primers specific for *esr1* (NM_152959), *esr2b* (AAH86848), *ensa* (NM_001045184), *sarcosine* (NM_198979), *fig alpha* (AY302567) and *zpc* (NM_131696) and *β-actin* (NM_131031) were designed using Primer Express software and the primer sequence for *esr2a* (AAH44349) was adapted as described [20]. The primer sets used are: *esr1*, F- 5′-CAGGACCAGCCCGATTCC-3′ and R- 5′-TTAGGGTACATGGGTGAGAGTTTG-3′; *esr2b*, F-5′-CGCTCGGCATGGACAAC-3′, R- 5′-CCCATGCGGTGGAGAGTAAT-3′; *esr2a*, F-5′-CTCACAGCACGGACCCTAAAC-3′, R- 5′-GGTTGTCCATCCTCCCGAAAC-3′; *β-actin*, F-5′-TGCCCCTCGTGCTGTTTT-3′, R- 5′-TCTGTCCCATGCCAACCAT-3′; *ensa*, F- 5′-TGGTGACCGGAGACCACAT-3, R- 5′-GCTGGTGAGGATGGTGTTTTTC-3′; *sarcosine*, F- 5′-GGAGATGCGCTACGCTTCAG-3′, R- 5′-GGCATCCTGGCAGCATTC-3′; *fig alpha*, F- 5′-CCCCGTTCACGTCAATATCG-3′, R- 5′-TGCGAGCTGTCGCCTCTT-3′; *zpc*, F- 5′-GGATGCCTTTAGGTTTCACAAGTT-3′, R- 5′-CCCGATTCTTAGCACTCACAGA-3′. Primer specificity was confirmed by standard curve analysis and *β-actin* expression was used to normalize expression of the three estrogen receptors. RNA from a zebrafish liver cell line (ZFL) was used as a calibrator in experiments using non-exposed adult fish. The stage before zygotic transcription (<3hpf) was used as a calibrator for the embryo experiments. Each experiment was carried out at least three times in triplicates.

### Statistical analysis

Numeric data from the expression experiments were analyzed in triplicates. Dispersion of the numbers and the symmetric distribution were analyzed using Student's t-test. Unpaired Student's t-test was used for the experiments in which ERs were activated with ligands. Probability of <0.05 was considered statistically significant. All graphs were plotted using the software GraphPad Prism 5.

### Bioinformatics analysis

Using the Clustal X program alignments of estrogen receptor beta LBDs of mouse, human and five fish species was pursued. Pairwise sequence comparisons of ERbeta subtypes were calculated. The accession numbers of ERbeta sequences of different species are: AAH86848 (zebrafish, *Danio rerio*, DrERβ1); AAH44349 (zebrafish, DrERβ2); AAD26921 (goldfish, *Carassius auratus*, CaERβ1); AAF35170 (goldfish, CaERβ2); ENSTRUT00000016675, pufferfish, *Fugu rubripes*, FrERβ1); ENSTRUT00000035144, pufferfish, FrERβ2); AAG16711, Atlantic croaker, *Micropogonias undulates*, MuERβ); AAG16712, Atlantic croaker, MuERγ); ENSORLG00000018012, medaka, *Oryzias latipes* OlERβ1); ENSORLT00000022182, medaka, OlERβ2); CAAO3949, house mouse, *Mus musculus*, MmERβ); NP_001428, human, *Homo sapiens*, HsERβ).

## Results

### *esr1* and *esr2* isofoms expressed in embryos and in adult zebrafish

**Figure 1:**
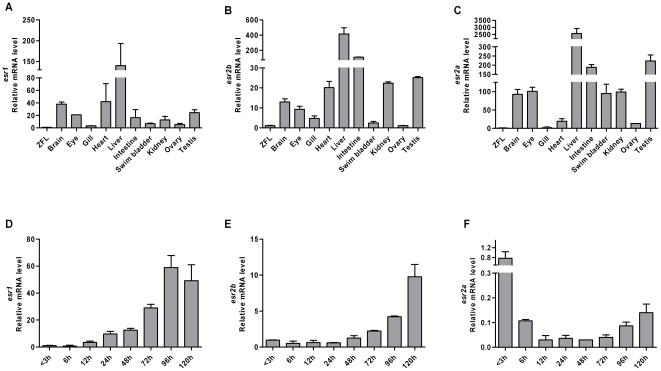
Expression levels of zebrafish estrogen receptors in organs, in embryos and in early larvae. The expression levels of *esr* genes are shown. (A) *esr1* expression in organs; (B) *esr2b* expression in organs; (C) *esr2a* expression in organs; (D) *esr1* expression in embryos and early larvae; (E) *esr2b* expression in embryos and early larvae; (F) *esr2a* expression in embryos and early larvae. Data are represented as mean ± SD of three independent experiments.

The real-time PCR methodology is a powerful tool to establish mRNA expression levels of specific molecules in organs and we used this methodology to evaluate the levels of the three estrogen receptors, *esr1*, *esr2a* and *esr2b* in zebrafish. Control experiments were pursued from early life stages to compare the stability of three reference genes in the real time PCR assays. The results showed that among the three control genes the *beta*-*actin* gene showed the most stable threshold values during the developmental stages (Figure S1). In adult fish the *beta-actin* Ct values was determined in liver, gut, eye, brain, testis and ovary in female and male adult fish. The expression of the *beta-actin* control gene showed Ct values in tissues between 17.3 and 23.4 in male fish and between 17.8 and 25.2 in female fish. The Ct values for control genes should be as stable as possible [21] and except for a higher Ct value in liver tissue the other tissues showed small variations under the conditions used (Figure S2). The expression of the estrogen receptor transcripts in the real time PCR showed that in absence of exposures and in a pool of five organs of female or male zebrafish, the *esr* transcripts were abundantly expressed albeit at different levels (Figure 1A–C). As expected, and shown by others, a high expression of *esr1* was detected in liver [11], [14], [18] but expression of *esr1* was also detected in organs such as brain, eye, heart and testis when compared to a zebrafish control liver cell line (Figure 1A). The two ERbeta transcripts, *esr2b* and *esr2a* were highly expressed in liver as well as in several of the other organs (Figure 1B–C). In brain the expression levels of *esr2b* represented approximately 3% of the expression levels found in liver. In testis, kidney and heart the amount of *esr2b* represented approximately 5% and in intestine approximately 25% of that found in liver (Figure 1B). Eye, gill, swim bladder and ovary showed very low levels of *esr2b* mRNA compared to the other organs (Figure 1B). The expression levels of *esr2a* in intestine and in testis were approximately 10% of the levels found in liver; the expression in brain, eye, swim bladder and kidney represented 5% and the *esr2a* expression in gill, heart and ovary showed lower expression levels (Figure 1C). ERbeta has been implicated in inflammatory disease [22] and the high expression levels of ERbeta genes in the intestine could suggest that the intestine may have an ERbeta related immunological role.

In comparison to the *esr1* mRNA in liver the expression levels were high in brain and testis (20–30% of the content in liver) whereas the expression levels of *esr1* in eye, gill, intestine, swim bladder, kidney and ovary were lower (Figure 1A). In ovary all three ERs showed a very low level of expression. To test the quality of the ovary mRNA we used primers specific for the germ cell transcription factor (*fig alpha*) [23] and for the egg envelope protein (*zpc*) [24] in real time assays. The results showed high expression of *fig alpha* and *zpc* in this organ (Figure S3) which strongly indicated that the mRNA utilized had not undergone degradation.

To determine the ER expression during the embryonic stages we collected eight stages, i.e. one stage prior to midblastula transition (MBT at 3 hpf), five stages during embryogenesis (6, 12, 24, 48 and 72 hpf) and two additional stages of early larvae (96 and 120 hpf). Using the primers that were previously utilized to detect mRNA content in adult tissue we showed the unique expression patterns of each ER gene type (Figure 1D–F). Onset of *esr1* expression occurred at the stage of zygotic control and accumulated from 12 hpf and in the following three stages (Figure 1D). The expression of *esr2b* also accumulated during development but with an onset of expression occurring 24 h later than that of *esr1* (Figure 1E). Both *esr1* and *esr2b* were expressed at the early larval stage (96 and 120 hpf). Expression of *esr2a* differed from the other two ERs and was decreased at 6 hpf, persisted at low levels at the stages of development and appeared again after hatching and showed expression at the early larval stages, 96 and 120 hpf (Figure 1F). The expression of all three ER subtypes was detected before MBT thus indicating their maternal origin [12], [20].

### Ligand dose response in embryos and in adult zebrafish

**Figure 2:**
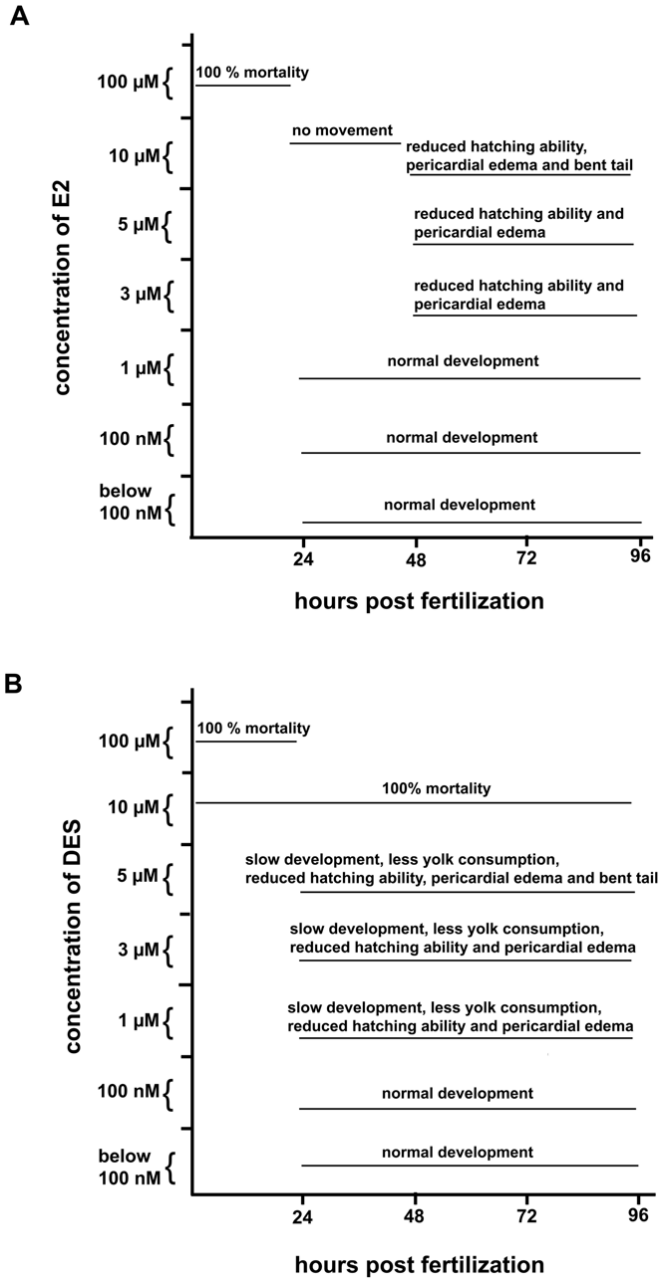
Effects of natural or synthetic ligand concentrations in early-life stages. The ligand dependent effects of different concentrations of E2 (A) or DES (B) during embryogenesis and early larval stages are shown. Following exposure of 7 hpf embryos the mortality rate and the visible phenotypes were monitored. Concentration of the ligands is plotted against the stage of development.

A regulatory role of the ER ligand, 17β-estradiol (E2) on target genes in mammalian cells has previously been described [25] and it has been shown that the *esr1* gene has a capacity to autoregulate itself in cells of liver origin both in mammals and in fish [18], [25]. We used three ligands in the real time assays, the natural ligand 17β-estradiol and the two synthetic ligands, diethylstilbestrol (DES) and nonylphenol (4-NP). The two latter ligands mimic the natural ligand in their capacity to induce ER target genes. In order to investigate the dose of the ligand causing an effect on the *esr1* expression level we pursued a dose response experiment using the three ligands. To determine the dose of ligand for the experiments, we used the concentrations 0, 0.1, 1, 10 nM, 0.1, 1, 3, 5, 10 and 100 µM for exposures. The parameters tested were a high survival rate and no apparent morphological traits of the embryos. The results showed that embryos and early larvae survived at the concentration 10 µM and 5 µM of E2 and DES respectively (Figure 2). The morphology of the embryos/early larvae was normal at exposure of 1 µM E2 (Fig. 2A) and at 100 nM DES (Fig. 2B) but effects such as heart edema and impaired hatching ability were seen at higher concentrations. In embryos the lowest concentration for an aberrant developmental phenotype caused by exposure to 4-NP (after 48 h) was 5 µM [26]. Lethality following in vivo exposure of adult fish for 48 h was tested on three female and three male fish at concentrations 1, 2, 5 and 10 µM of ligand. The fish survived at the concentration 2 µM E2. At 5 µM E2 all three female fish survived but all male fish died. A similar exposure experiment using DES and 4-NP showed that all fish died if exposed to 5 µM of ligand, whereas all fish survived at 2 µM (not shown). Concentrations in between 2 and 5 µM were not tested. In the following exposure experiments we used 1 µM and 100 nM of ligand.

### Ligands increase ER expression in embryos and in early larvae

**Figure 3:**
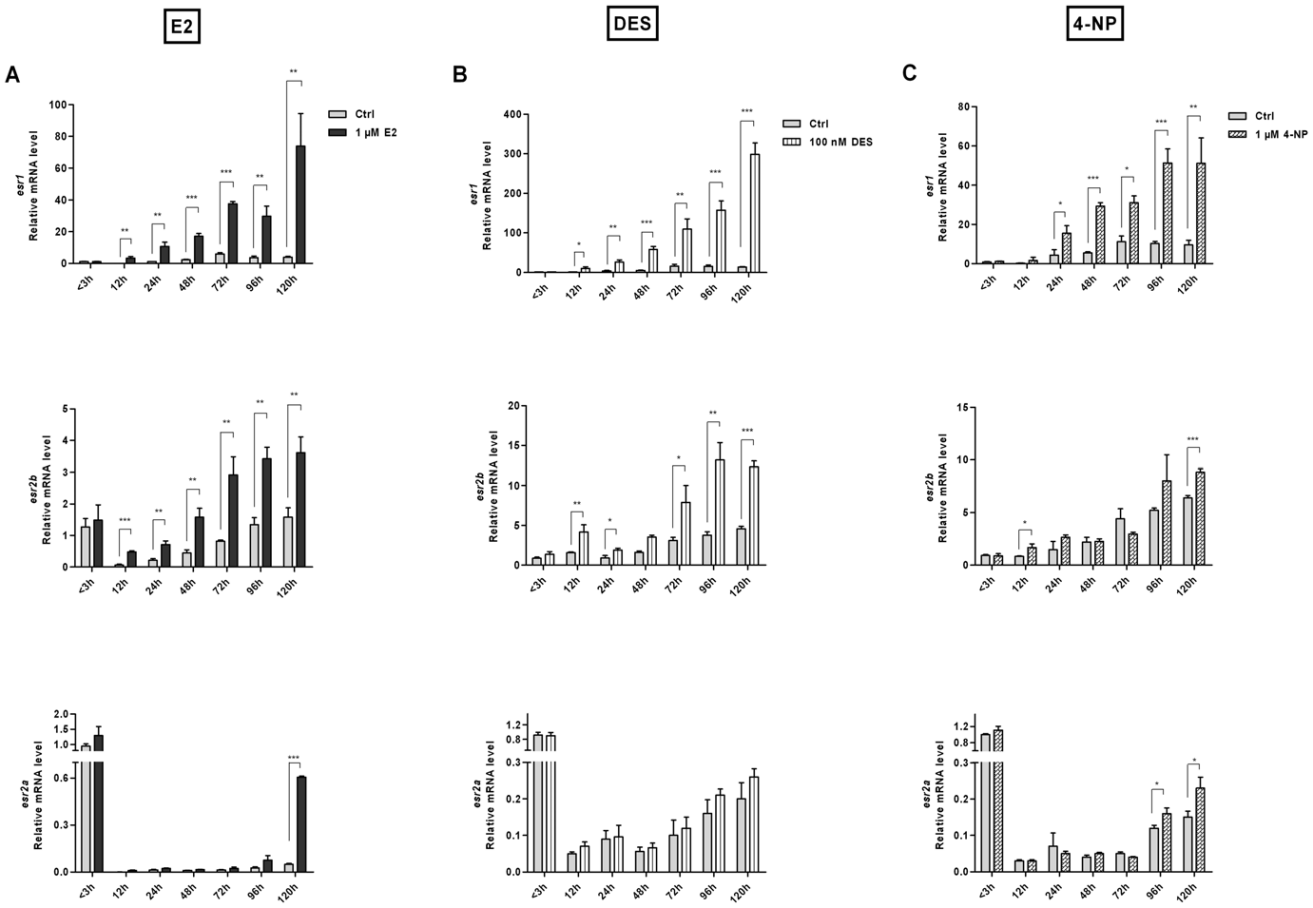
Effects of natural or synthetic ligands on *esr* expression levels in early-life stages. Expression levels of *esr* genes in embryos and early larvae in absence (ctrl) and presence of ligand. Embryos were exposed to E2 (1 µM), DES (100 nM), 4-NP (1 µM) or solvent control (0.1% ethanol) one-hour post fertilization until processed for qPCR. Transcript levels after treatment with E2 (A), DES (B) or 4-NP (C) are shown. Data are represented as mean ± SD of three independent experiments. Statistical significance was measured by Students's *t* test and asterisks (* *p*<0.05; ** *p*<0.01; ***p<0.001) indicate significant differences between the control and ligand treated groups for each developmental stage.

Estrogen effects on the expression levels of ERs in zebrafish early life have not been well characterized and except for *esr2a* expression levels [20], and *esr* expression at stages preceding 48 hpf, [27] there is sparse information on the quantitative expression levels of ER subtypes during development and in the early larvae. To investigate a possible regulatory role of the natural ligand in zebrafish embryos, we exposed the embryos one-hour post fertilization to E2 or vehicle (0.1% ethanol). Exposed embryos showed *esr1* mRNA expression during development and in the stages of early larvae (Figure 3A). At the lower concentration (100 nM) the strong agonist ligand, DES yielded a similar result on *esr1* (Figure 3B). An increased expression of *esr1* was also detected following 4-NP exposures (Figure 3C). These data clearly show sensitivity of the expression levels of the *esr1* gene in presence to the natural and of synthetic ligands.

Since *esr1* expression was increased in presence of E2, DES and 4-NP during early stages, we reasoned that also the gene products of ERbeta might respond to these ligands. As shown in Figure 3A, the expression of *esr2b*, but not *esr2a* was affected by E2 at the stages between 12 and 72 hpf. At the two larval stages (96 and 120 hpf) however, an increase could be detected of both beta gene products in presence of E2 (Figure 3A). As DES had a stronger effect in the dose response study and also has a higher binding affinity for ERs [15], [28] we used the lower concentration of DES (100 nM) in the exposures of the zygotes. Figure 3B and 3C show an induction of *esr2b* by DES; in a similar set up, no significant effect was detected after exposure to 4-NP. As both DES and 4-NP were potent inducers of *esr1* the low expression of *esr2b* following the exposures was not due to lower uptake of the ligand. The low *esr2a* mRNA levels during development were not altered by any of the ligands. At the early larval stages an induction of the *esr2a* expression was detected following exposure of ligand. Our results show a clear difference in expression levels between the two beta gene products in response to ligand exposures at the early life stages.

### Ligands modulate expression levels of ERs in liver, brain and gonads of female and male fish

**Figure 4:**
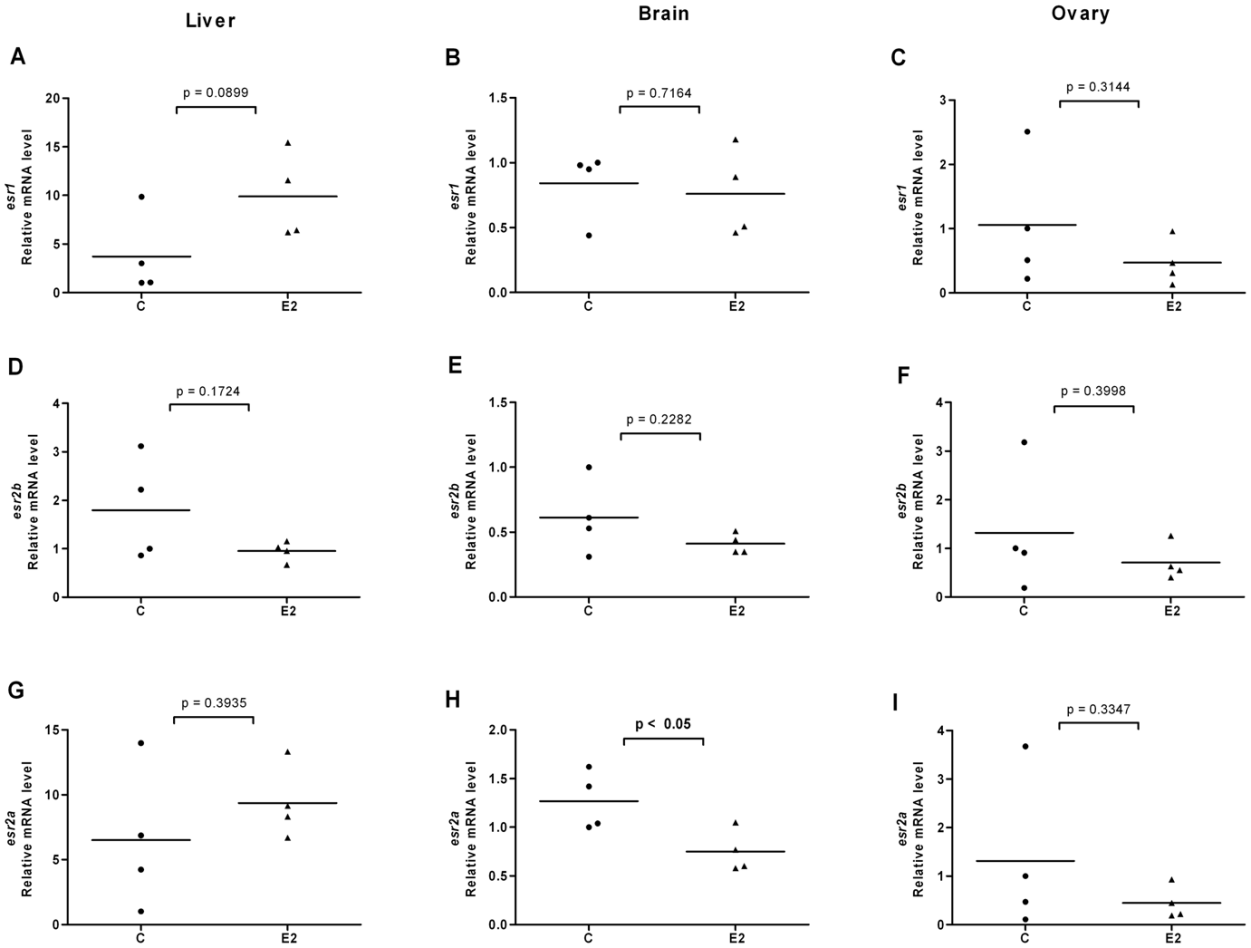
Effects of 17β-estradiol on *esr* expression in liver, brain and ovary of female fish. Expression levels of *esr1* (A–C), *esr2b* (D–F) and *esr2a* (G–I) in liver, brain and ovary of female zebrafish following exposure to E2 (1 µM). Ethanol (0.1%) was used as solvent control and adult females were exposed to E2 or solvent control for 48 h and mRNA levels of *esr1*, *esr2b* and *esr2a* were quantified. After normalization with *beta-actin* the expression levels of individual fish of both groups are shown. The mean value is indicated by a line.

**Figure 5:**
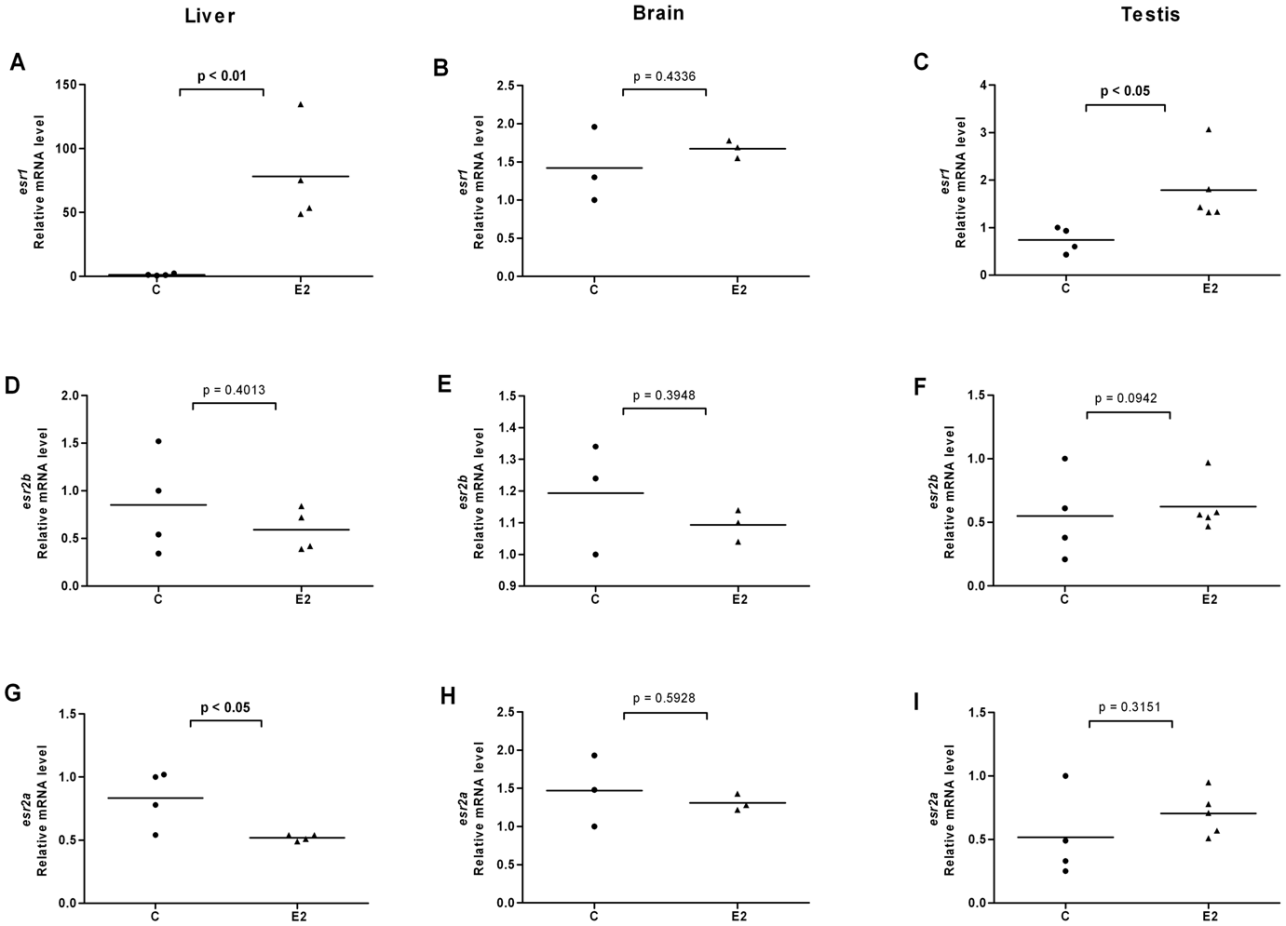
Effects of 17β-estradiol on *esr* expression in liver, brain and testis of male fish. Expression levels of *esr1* (A–C), *esr2b* (D–F) and *esr2a* (G–I) in liver, brain and testis of male zebrafish following exposure to E2 (1 µM). Normalization and mean values were calculated as in figure 4.

**Figure 6:**
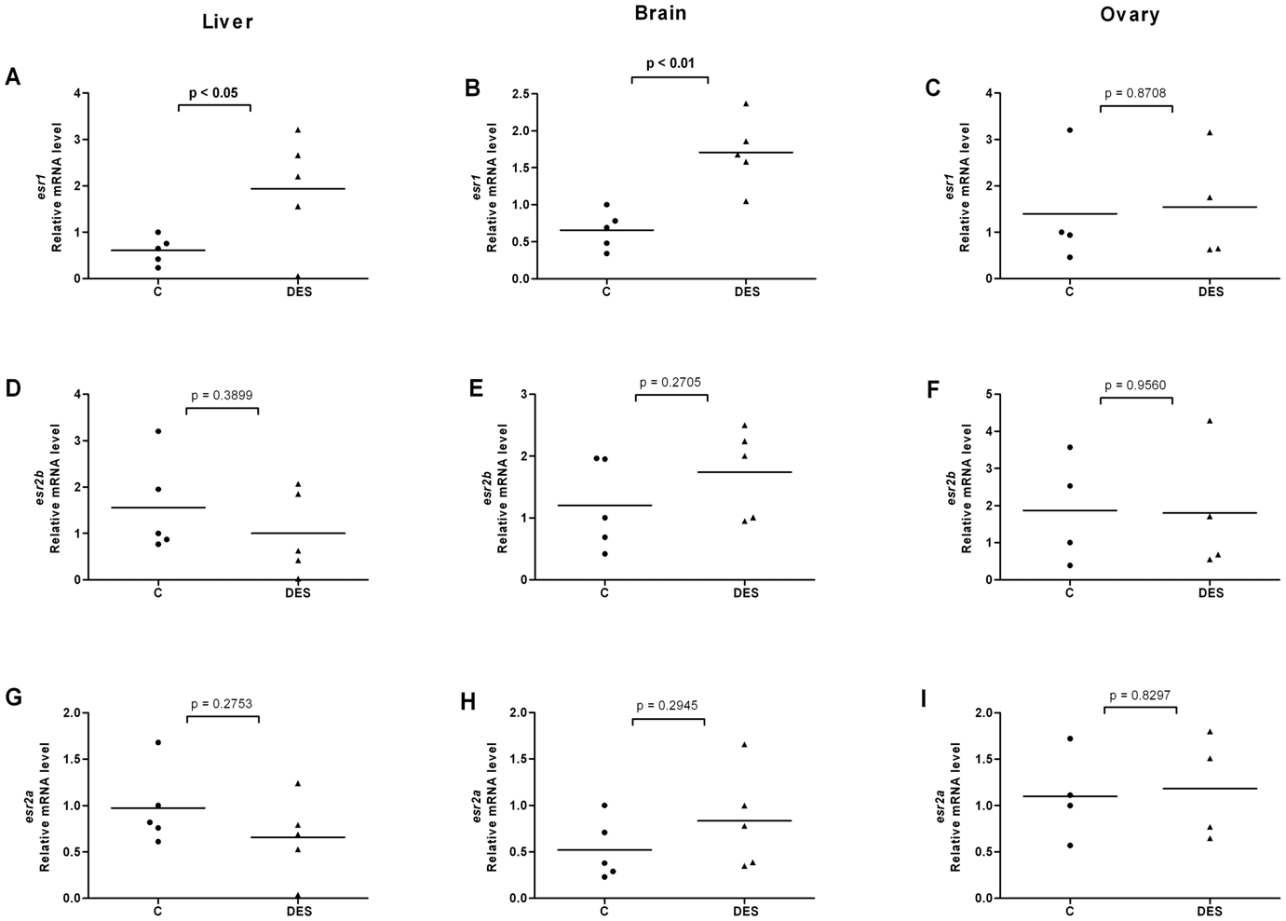
Effects of diethylstilbestrol on *esr* expression in liver, brain and ovary of female fish. Expression levels of *esr1* (A–C), *esr2b* (D–F) and *esr2a* (G–I) in liver, brain and ovary of female fish following exposure to DES (1 µM).

**Figure 7:**
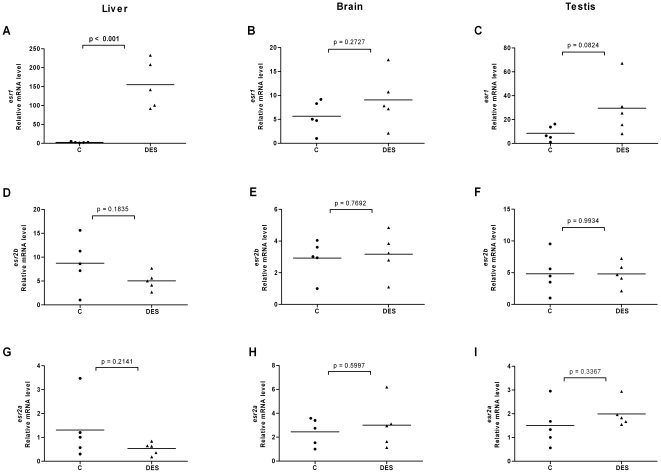
Effects of diethylstilbestrol on *esr* expression in liver, brain and testis of male fish. Expression levels of *esr1* (A–C), *esr2b* (D–F) and *esr2a* (G–I) in liver, brain and testis of male fish following exposure to DES (1 µM).

**Figure 8:**
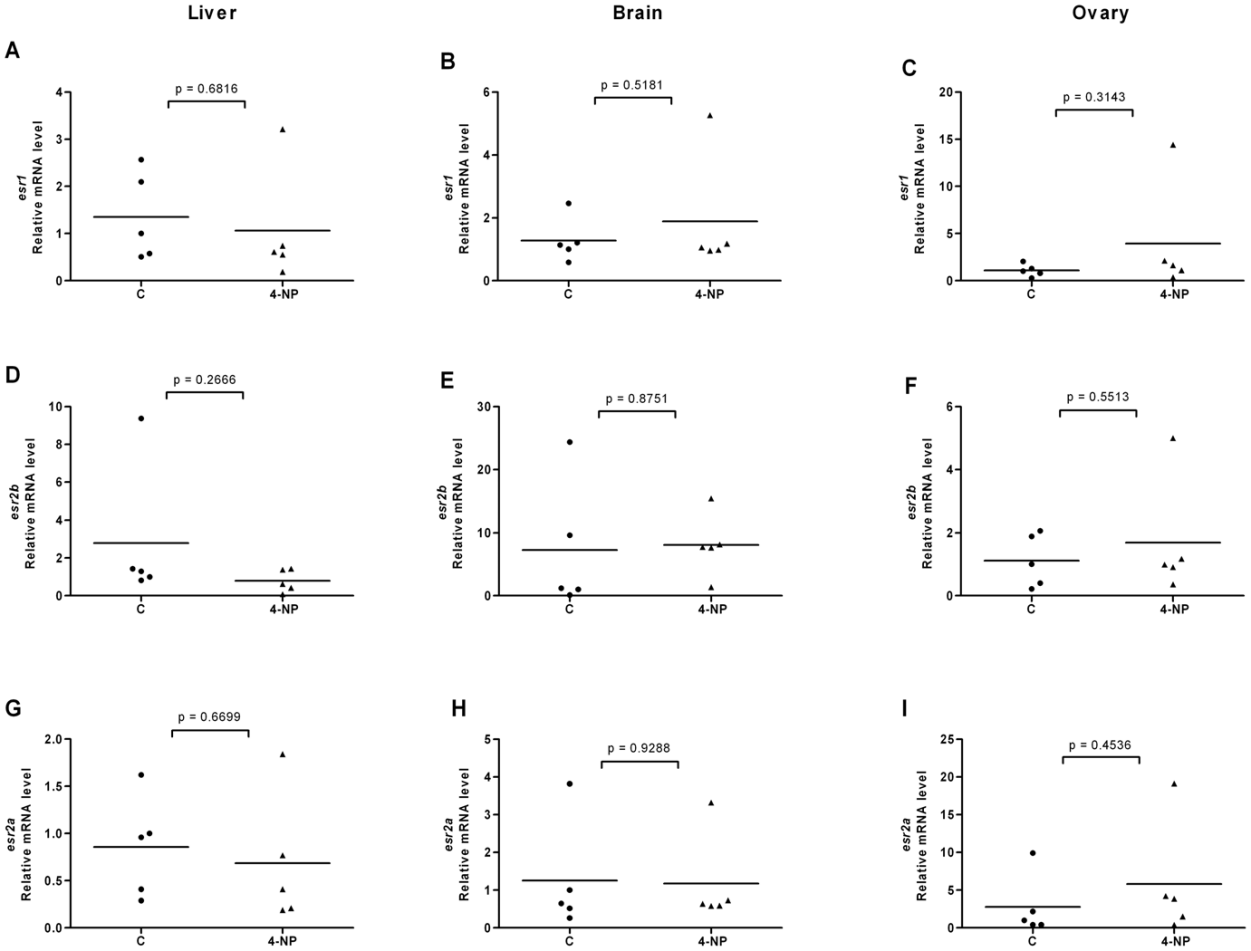
Effects of 4-nonylphenol on *esr* expression in liver, brain and ovary of female fish. Expression levels of *esr1* (A–C), *esr2b* (D–F) and *esr2a* (G–I) in liver, brain and ovary following exposure to 4-NP (1 µM).

**Figure 9:**
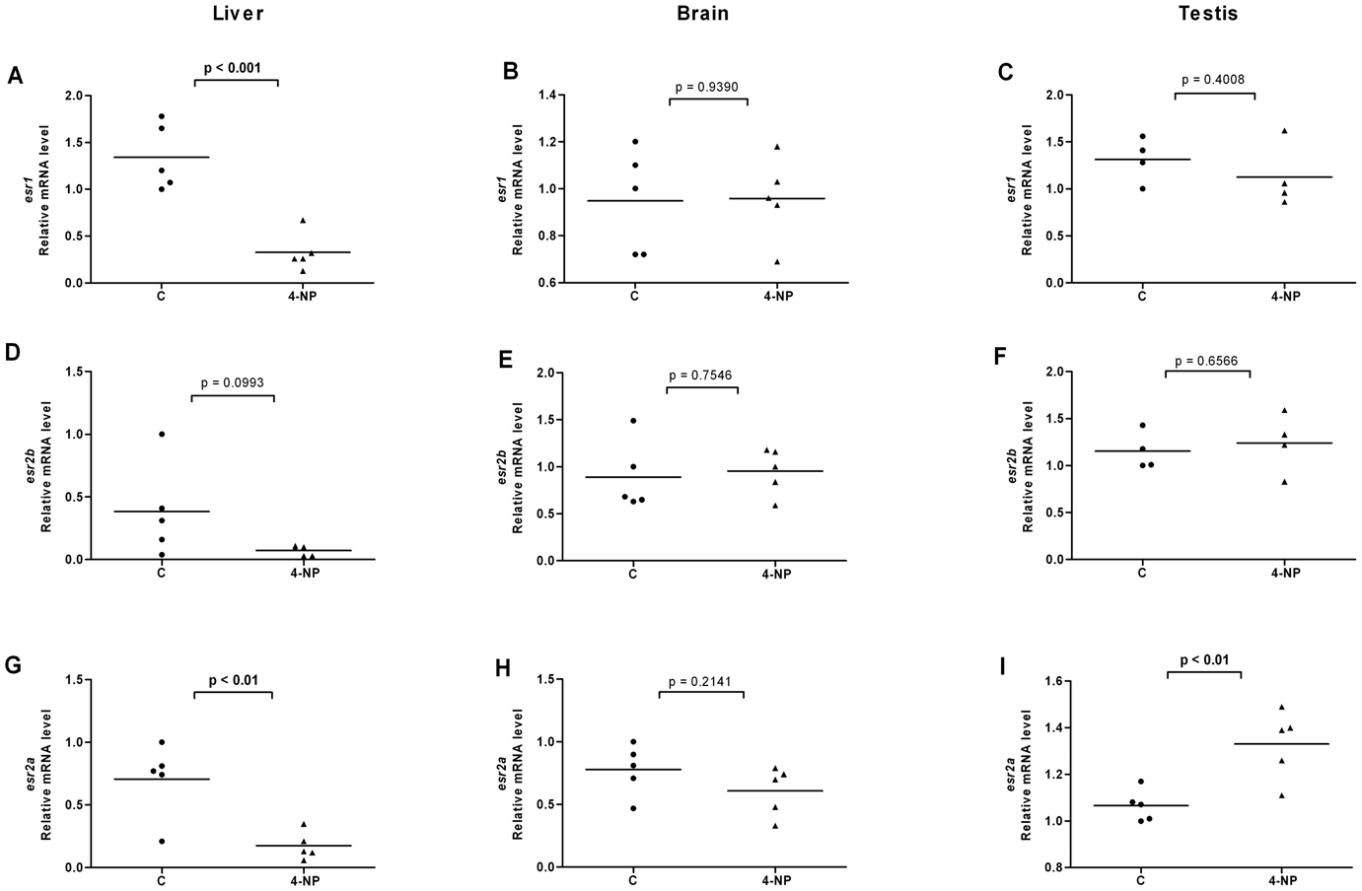
Effects of 4-nonylphenol on *esr* expression in liver, brain and testis of male fish. Expression levels of *esr1* (A–C), *esr2b* (D–F) and *esr2a* (G–I) in liver, brain and testis following exposure to 4-NP (1 µM).

In order to investigate the effects of ligands on ER expression in tissues of adult zebrafish and in particular in tissues involved in estrogen metabolism and signaling, we exposed fish to E2, DES or 4-NP. We isolated organs of individual female and male fish, isolated the mRNA and compared the ER expression levels in each individual organ. We utilized the same *esr2* specific primers that were earlier used for real time PCR on target cDNAs derived from stages of development and of early larvae. The results for *esr* expression levels in liver, brain, ovary and testis are shown in Figure 4, Figure 5, Figure 6, Figure 7, Figure 8 and Figure 9. Exposure to E2 of female or male fish increased the expression of *esr1* in liver and in testis but not in brain or ovary (Figures 4A–C, 5A–C). Furthermore, in male fish we found increased levels of *esr1* in eye and intestine following E2 exposure (not shown). Exposure to DES showed similar results as with E2 i.e. *esr1* expression was increased in liver, but, in addition, increased expression levels of *esr1* were seen in brain of female fish (Figures 6A–B, 7A). In contrast to E2 and DES, 4-NP strongly decreased *esr1* expression in liver which is in agreement with the decrease in liver reported for three other fish species [29] [30] [31].

Changes in the expression of *esr2b* could not be detected in the tissues tested in female or male zebrafish. However expression of *esr2a* showed ligand specific changes in some tissues. In female fish the natural ligand but not the synthetic ligands had effects on transcript levels and increased *esr2a* expression in liver and decreased *esr2a* expression in brain (Figure 4G–H). The expression levels of *esr2a* following E2 exposure decreased in liver of male fish. The exposure to 4-NP, but not DES, resulted in a significant decrease of *esr2a* expression in liver of male fish (Figure 7G, 9G). In testis exposure to 4-NP, but not E2 or DES significantly increased the expression levels of *esr2a* (Figure 5I, 7I, 9I). Similar data was recorded in testis from another zebrafish strain (AB strain) following *in vivo* exposure to 4-NP (not shown).

In eye or intestine of female or male fish, *esr2a* expression did not change following exposure to 4-NP (not shown).

In brain of rats there are genes reported to be targets of 4-NP [32] and we identified in the databank two orthologues of these genes, *sarcosine* and *ensa* of zebrafish. To test these two orthologues in our zebrafish system, we designed primers specific to the zebrafish *sarcosine* and *ensa* genes, and used these primers on cDNA derived from brain of male zebrafish exposed to 4-NP. 4-NP had a stimulatory effect on *sarcosine* expression and a slightly repressive effect on the *ensa* gene in brain although with low significance (Figure S4). This might indicate a similar response to 4-NP in brain of rat and zebrafish.

### The sequence conservation of the esr2a and esr2b

Only one ERbeta gene has been identified in mammalian species. In the teleost lineage several genes including ERbeta (but not ERalpha) have undergone gene duplication [33], [34] generating two genes in many fish species including zebrafish. We hypothesized that the human ERbeta structurally may have similarities to one of the two ERbeta proteins in zebrafish. We used bioinformatics to align one of the conserved domains, the ligand binding domain (LBD) of ERbeta in human and in mouse to the corresponding regions of esr2b and esr2a in zebrafish and in four other fish species that harbor two types of ERbeta. The results showed that i) the LBDs of mammalian ERbeta are approximately 70% homologous to esr2a and esr2b and ii) in this region the homology between the two ERbeta peptides in zebrafish is 76% (Table 1). The differences in the primary sequence of ERbeta LBD (not shown) do not indicate that human ERbeta is more closely related to specifically any one of the two subtypes in zebrafish.

**Table 1:** Amino acid conservation in the ERbeta ligand binding domains.

	Hs ERβ	Mm ERβ	Mu ERβ	Mu ERγ	Ca ERβ1	Ca ERβ2	Ol ERβ2	Ol ERβ1	Fr ERβ2	Fr ERβ1	Dr ERβ2	Dr ERβ1
**Dr ERβ1**	72%	71%	82%	73%	74%	95%	72%	100%	74%	79%	76%	100%
**Dr ERβ2**	71%	69%	77%	86%	93%	76%	89%	76%	87%	75%	100%	76%

Calculations of the homologies in the ligand binding domains. Zebrafish and four other fish species were compared with the corresponding regions in human and mouse. Dr, zebrafish; Ca, goldfish; Fr, pufferfish; Mu, Atlantic croaker; Ol, medaka; Mm, house mouse; Hs, human.

## Discussion

In this study we measured mRNA levels of the genes encoding the estrogen receptors in zebrafish. Expression levels of *esr2a* during developmental stages 3–72 hpf have previously been reported [20] and here we show expression levels of all three receptors in adult tissues, in embryos and in early larvae. Genes such as *vitellogenin* [35], *aroB* [36] and *esr1* [18] have previously been reported as target genes of ER in zebrafish and here we show data that indicate that *esr2a* and *esr2b* genes are direct or indirect targets of ER ligands.

### Ligands alter the *esr* gene expression *in vivo*

It has previously been reported that the *esr1* promoter functions as an E2 induced target in liver tissue of fish [18]. In mammalian species a vast number of genes have been identified that respond to ER in presence of ligand [25] but in fish only a limited number of such genes have been identified i.e. vitellogenins [37], aromatase [31], [38] and esr1 [18]. The zebrafish has become a suitable model system for assessing synthetic ligands that function as disrupters of the endocrine system.

The two ERbeta genes in zebrafish have different levels of expression depending on tissue context. The existence of two ERbeta genes in zebrafish (and in a number of other fish species) is an example of gene duplication and which put focus on possible functional differences between *esr2a* and *esr2b*. The two ERbeta genes are conserved but their primary sequences are not identical (Table 1). There are examples of other paralogue genes in zebrafish showing differences in expression patterns [39]–[41] and with possible differences in function. In other model systems it has been shown that paralogues can have different functions, for instance in case of the maturation switch of globin genes [42]. The low expression level of the zebrafish *esr2a* gene in control and exposed embryos (Figure 1F) indicates that the two ERbeta proteins may have diverse functions during development. ER expression has been shown during early life stages in specific cell types of pectoral fin buds, in cells of the hatching gland, in brain cells and in neuromasts [20], [43], [44]. Increased expression of *esr1* and *esr2b* following ligand exposure for two days (Figure 3A–B) indicates that these genes are under regulatory control.

Although the PCR methodology is highly sensitive, the efficiency by which a ligand enters a particular organ following in vivo exposure may be a problem due to low penetration efficiency. For solving these issues additional methodology will be needed for the characterization of *esr* genes as target genes of estrogenic compounds. Another limitation with the PCR methodology is the low number of well characterized control genes. A larger number of control genes may facilitate detection of small changes in expression levels of the gene of interest. A control gene that shows stable Ct values in different tissues and/or during the developmental stages will increase the accuracy [21]. Another factor to consider is the amount of starting material in the PCR assays as this may influence the Ct values.

### 4-nonylphenol, an estrogen mimicking regulator?

Alkylphenol ethoxylates (APEs) have been used for 50 years in industrial manufacturing and nonylphenol is the major breakdown product of APEs. Nonylphenol, a xenoestrogen first detected in sewage of water treatment plants in 1984 [17] has been extensively studied as one of the most potent environmental chemicals (EDCs) [13], [45], [46]. There are accumulated data on negative effects of human exposure [47] and in animal models endocrine and reproductive effects of nonylphenol have been shown such as inhibited testicular growth [48], [49], lower semen volume [50] and lower fertility [51]. In zebrafish embryos it has been shown that 4-NP mediates neurotoxic effects [26] and aberrant effects on specific cell types [52].

4-NP has been shown to induce target genes in a number of arrays and in other systems. This ligand is efficient in the induction of the vitellogenin gene, one of the biomarkers that frequently has been used to detect estrogenic effects in male fish [51], [53]. In microarray systems a number of genes have been identified that are differentially expressed following exposure to 4-NP [53]; in fact the number of 4-NP induced genes exceeds that following exposure to the natural ligand in embryonic zebrafish [54]. In a mammalian array study [32] the 4-NP ligand was shown to regulate *sarcosine* and *ensa* genes, a result we were able to reproduce in brain of male zebrafish (although not with high significance; Figure S4).

There are differences in the potency by which specific ligands exert their effects in female and male fish. This difference may be explained by sex differences in the levels of circulating 17β-estradiol, which perhaps is the cause for the less distinct effects we see on *esr* gene expression in female fish following ligand exposure (for instance in liver, Figure 4, 5, 6, 7, 8, 9). However, following exposure of ligand the female and male fish can show opposite effects in levels of ER expression which may indicate a sexually divergent mechanism. Regulation of *esr2a* expression in brain of female but not male fish (Fig. 4H), and *esr2a* expression in testis but not in ovaries (Fig. 9I) are examples of sex specific control of ERs. Further studies to evaluate sex specific expression levels of *esr* genes may reveal novel aspects of ER signaling in female and male zebrafish.

A general view that environmental synthetic chemicals are acting as ‘weak estrogens’ can possibly be challenged by the potency they seem to have. 4-NP has the capacity to disrupt events in adult organs as well as during development, and these effects are detected at concentrations that are not affecting survival or the visual phenotype. Adult zebrafish and zebrafish embryos are well suited as *in vivo* models for studying molecular effects in individual fish that have been exposed to 4-NP.

## Supporting Information

Figure S1 Expression of three different control genes during development. Quantitative real time PCR was performed on embryos and early larvae treated with solvent control (0.1% ethanol) and E2 (1 µM). Ct values of *beta-actin* (A), *18s rRNA* (B) and elongation factor 1 alpha (*ef1α*) were calculated (C). Data represents mean Ct ± SD of three independent experiments.
(0.34 MB TIF)

Figure S2 Expression of *beta actin* in organs. Average Ct values of *beta actin* in different tissues of adult male and female zebrafish following solvent control and E2 exposures. Tissues from three or four adult female and male fish were collected and processed for qPCR as described in materials and methods. Mean Ct values ± SD of *beta-actin* in solvent control (0.1% ethanol) and E2 exposed groups are shown.
(0.03 MB DOC)

Figure S3 Expression of two female specific genes in ovary. Relative mRNA levels of germ cell transcription factor, *fig alpha* (A) and egg envelope protein, *zpc* (B) in organs of adult fish are represented. Data are represented as mean ± SD of three independent experiments.
(0.34 MB TIF)

Figure S4 Expression of two genes in male brain following 4-NP exposure. Expression of *sarcosine* (A) and *ensa* (B) in brain of adult male zebrafish following exposure of 4-NP. Brain of five adult males exposed to 4-NP or solvent control was processed for qPCR as described in materials and methods. After normalization with *beta actin* the expression levels of individual fish of both groups are shown. The mean value is indicated by a line.
(0.32 MB TIF)
