# Supplementary figures and images for: Levels of 17β-Estradiol Receptors Expressed in Embryonic and Adult Zebrafish Following In Vivo Treatment of Natural or Synthetic Ligands
Source: PLoS One. 2010 Mar 12;5(3):e9678. doi: 10.1371/journal.pone.0009678 (PMC2837374; doi:10.1371/journal.pone.0009678)

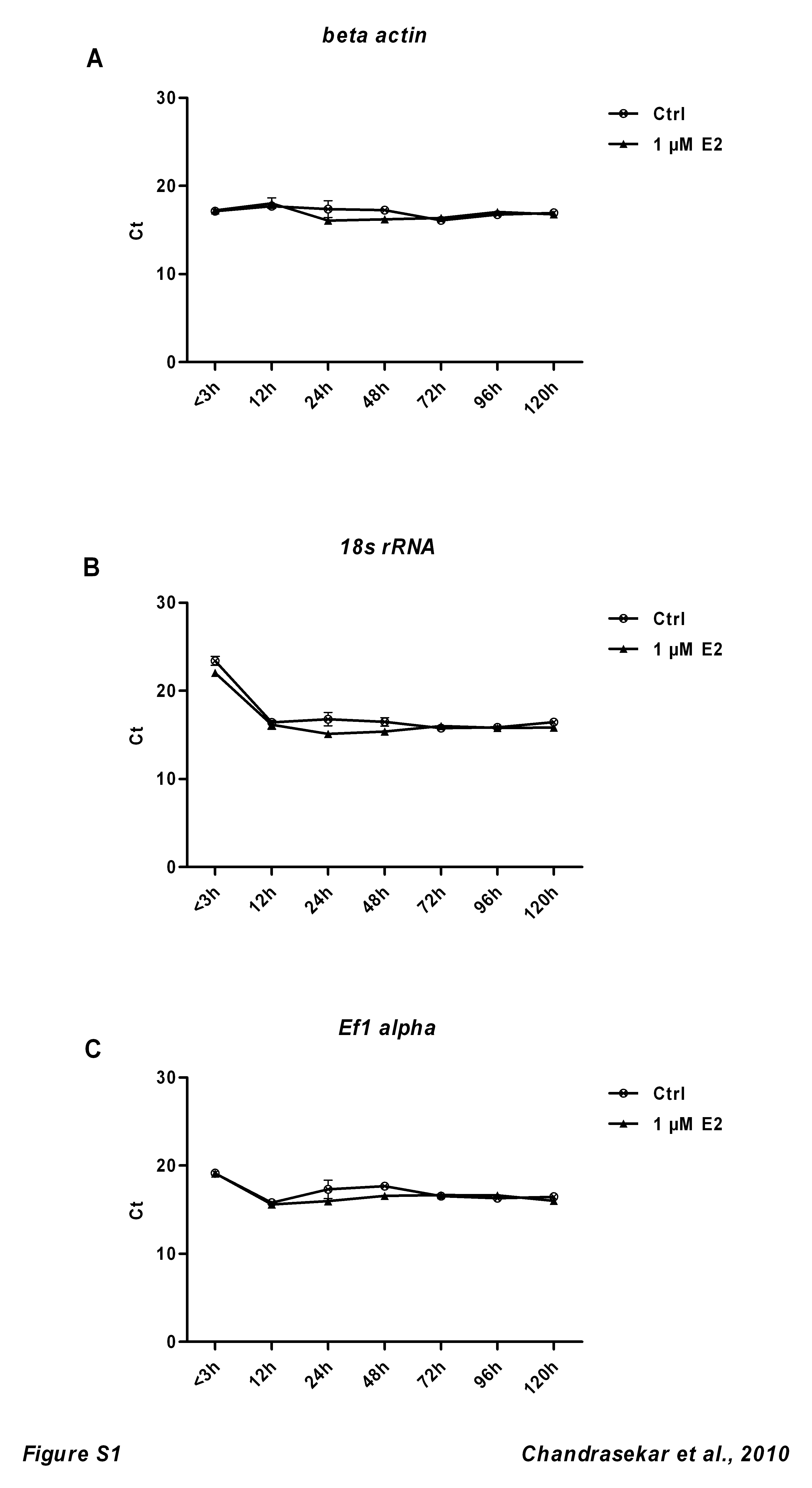

Supplement: Figure S1 — Expression of three different control genes during development. Quantitative real time PCR was performed on embryos and early larvae treated with solvent control (0.1% ethanol) and E2 (1 µM). Ct values of beta-actin (A), 18s rRNA (B) and elongation factor 1 alpha (ef1α) were calculated (C). Data represents mean Ct ± SD of three independent experiments. (0.34 MB TIF) [file pone.0009678.s001.tif]

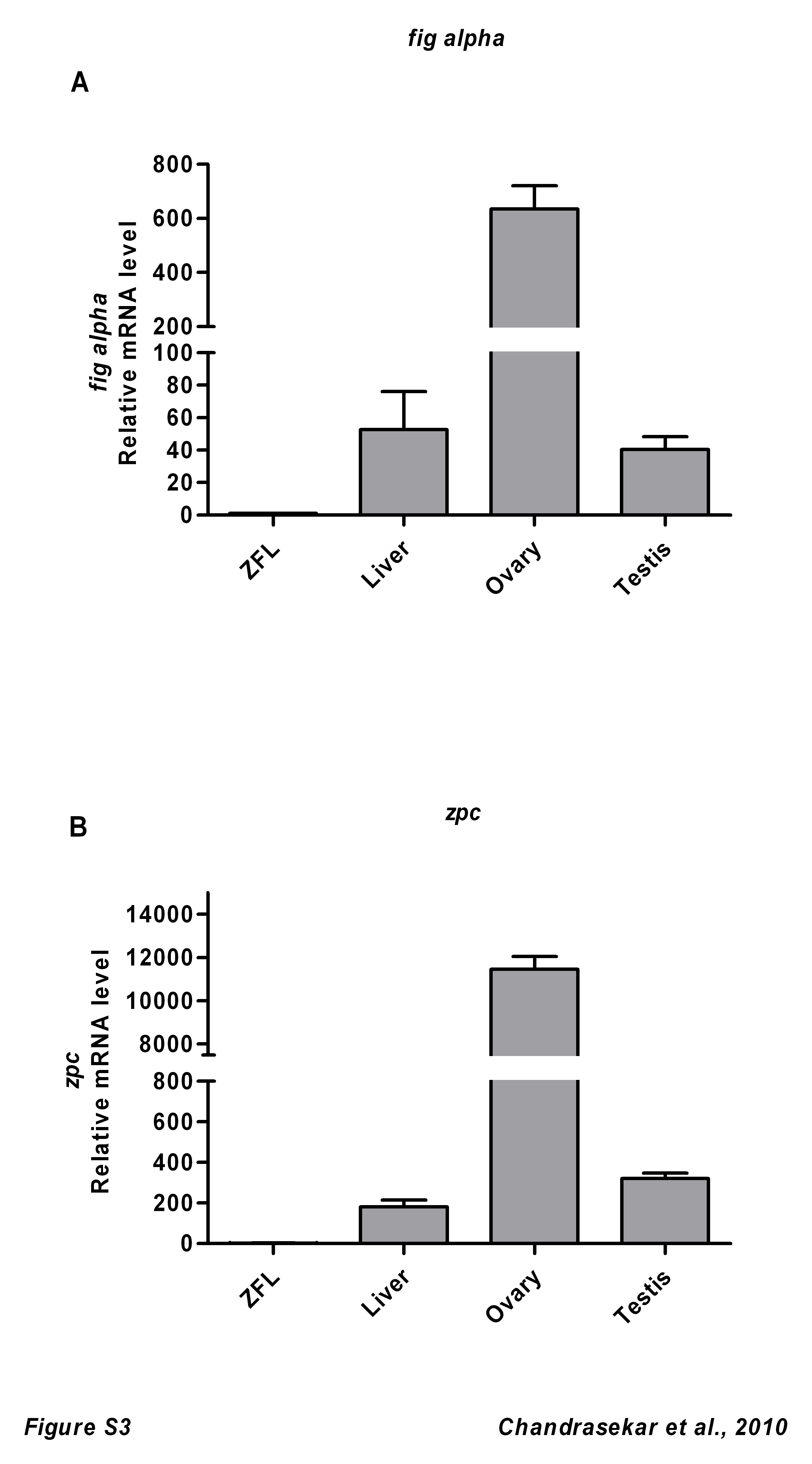

Supplement: Figure S3 — Expression of two female specific genes in ovary. Relative mRNA levels of germ cell transcription factor, fig alpha (A) and egg envelope protein, zpc (B) in organs of adult fish are represented. Data are represented as mean ± SD of three independent experiments. (0.34 MB TIF) [file pone.0009678.s003.tif]

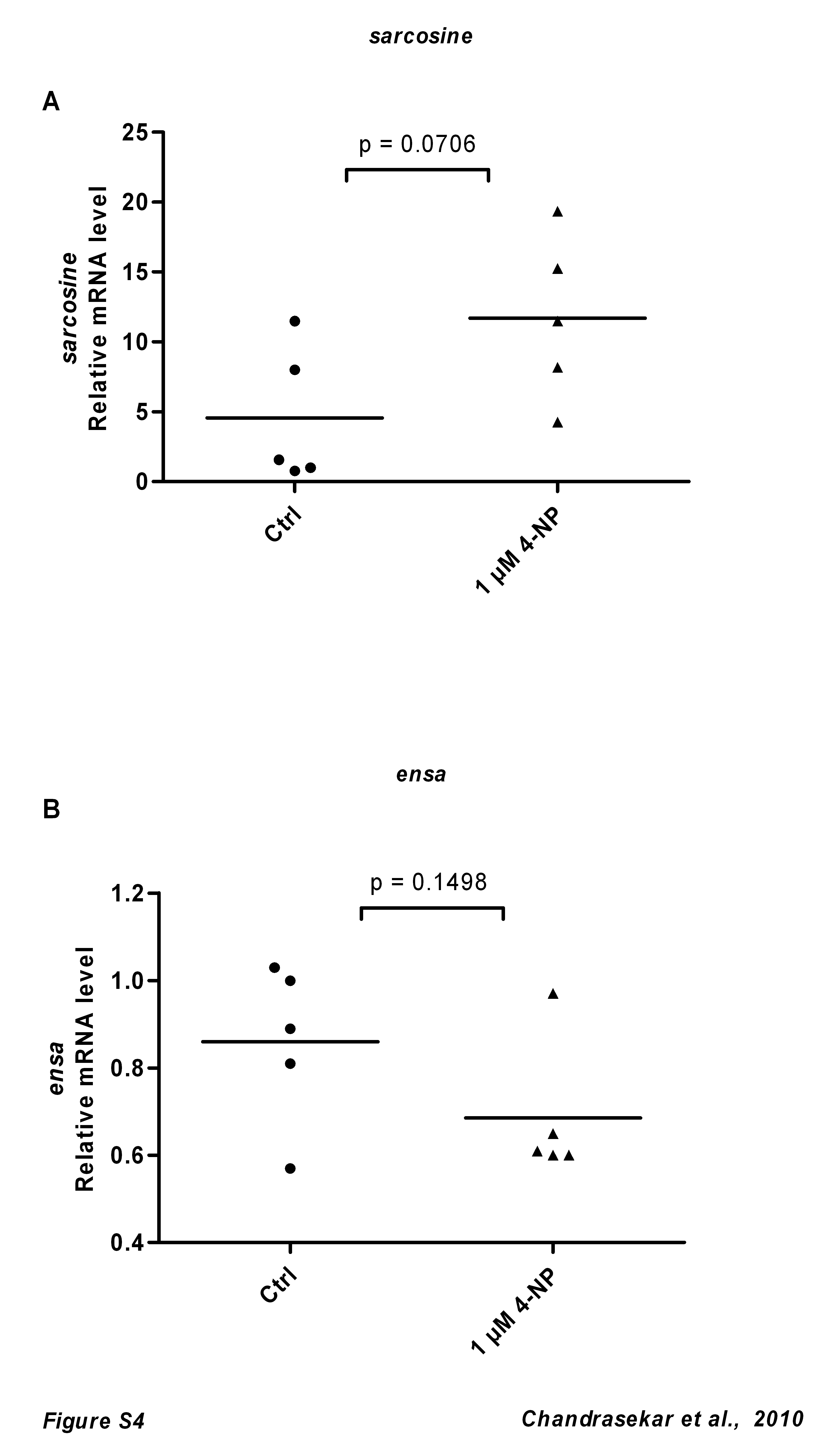

Supplement: Figure S4 — Expression of two genes in male brain following 4-NP exposure. Expression of sarcosine (A) and ensa (B) in brain of adult male zebrafish following exposure of 4-NP. Brain of five adult males exposed to 4-NP or solvent control was processed for qPCR as described in materials and methods. After normalization with beta actin the expression levels of individual fish of both groups are shown. The mean value is indicated by a line. (0.32 MB TIF) [file pone.0009678.s004.tif]
