# Supplementary material for: Levels of 17β-Estradiol Receptors Expressed in Embryonic and Adult Zebrafish Following In Vivo Treatment of Natural or Synthetic Ligands
Source: PLoS One. 2010 Mar 12;5(3):e9678. doi: 10.1371/journal.pone.0009678 (PMC2837374; doi:10.1371/journal.pone.0009678)
Supplement: Figure S2 — Expression of beta actin in organs. Average Ct values of beta actin in different tissues of adult male and female zebrafish following solvent control and E2 exposures. Tissues from three or four adult female and male fish were collected and processed for qPCR as described in materials and methods. Mean Ct values ± SD of beta-actin in solvent control (0.1% ethanol) and E2 exposed groups are shown. (0.03 MB DOC) [file pone.0009678.s002.doc]

Figure S2:

| **Ct values of *beta actin* ± S.E.M** | | | | | |
| --- | --- | --- | --- | --- | --- |
| Male | | | | | |
|  | Liver | Gut | Eye | Brain | Testis/Ovary |
| Ctrl | 23.4±0.57 | 20.1±0.74 | 20.6±0.41 | 20.6±0.25 | 17.3±1.03 |
| 1 µM E2 | 23.3±0.25 | 19.9±0.59 | 20.6±0.18 | 21.5±0.77 | 16.8±0.25 |
| Female | | | | | |
| Ctrl | 25.2±0.67 | 21.3±1.21 | 20.6±0.2 | 21.4±0.39 | 17.8±1.46 |
| 1 µM E2 | 24.9±0.34 | 20.8±1.07 | 21.0±0.08 | 21.3±0.38 | 17.5±0.54 |
